# Identifying and validating the educational needs to develop a Celiac Self-Care System

**DOI:** 10.1186/s12875-023-02076-8

**Published:** 2023-06-14

**Authors:** Mostafa Langarizadeh, Pakzad Rahmati, Shahram Yousefpour Azari, Fatemeh Sarpourian, Mohammad Javad Sayadi, Mohammad Hossein Langarizadeh, Seyed Ali Fatemi Aghda

**Affiliations:** 1 Department of Health Information Management, School of Health Management and Information Sciences, Iran University of Medical Sciences, Tehran, Iran; 2 Department of Internal Medicine, Faculty of Medicine, Shahid Beheshti University of Medical Sciences, Tehran, Iran; 3 Ph.D. Candidate of Health Information Management, Student Research Committee, Department of Health Information Technology, School of Health Management and Information Sciences, Shiraz University of Medical Sciences, Shiraz, Iran; 4 Department of Computer Sciences, Faculty of Mathematics Sciences, Shahid Beheshti University, Tehran, Iran

**Keywords:** Minimum data, Self-care, Celiac disease, Data requirements, Educational needs, Mobile apps, Application program

## Abstract

**Background:**

Celiac disease is a major public health problem in many countries, including Iran. Considering the disease’s exponential spread throughout the world and its risk factors, identifying the educational priorities and minimum data required to control and treat the disease is of great significance.

**Methods:**

The present study was conducted in two phases in 2022. In the first phase, a questionnaire was developed based on the information obtained from a review of the literature. Later, the questionnaire was administered to 12 pundits in the fields of nutrition (*n* = 5), internal medicine (*n* = 4), and gastroenterology (*n* = 3). As a result, the necessary and important educational content was determined for developing the Celiac Self-Care System.

**Results:**

According to the experts’ viewpoints, the educational needs of patients were classified into nine categories of demographic information, clinical information, long-term complications, comorbidity, tests, medications, dietary recommendations, general recommendations, technical capabilities as well as 105 subcategories.

**Conclusions:**

Due to the increased prevalence of Celiac disease and the lack of an established minimum set of data, determining the required educational information is of great importance at the national level. Such information could be useful in implementing educational health programs to raise the public level of awareness. In the field of education, such contents can be employed in planning new technology based on mobile phones (mobile health), preparing registries, and producing widely used content.

**Supplementary Information:**

The online version contains supplementary material available at 10.1186/s12875-023-02076-8.

## Background

Celiac Disease (CD), caused by the interaction of gluten with the immune system, genetic, and environmental facto, has turned into a public health problem and one of the most devastating gastrointestinal diseases worldwide due to its increased rate of prevalence [1, 2]. Currently, medical nutrition therapy including a gluten-free diet is the only accepted treatment for CD. Gluten is a water-insoluble protein (gliadins, glutenin, hordein, and secolin) that exists in triticale, rye, wheat, and barley [3, 4]. Although diet adherence reduces the consumption of vitamins (group B), fiber, etc., compliance with this diet is not easy for the patients considering the wide variety of foods [4–6].

During the last two decades, CD has been commonly reported in North Africa, the Middle East, India, and Pakistan [7]. In Iran, various studies reported the disease prevalence at about 2–3 percent with a higher rate in the central and western regions than in other regions [8, 9]. The disease complications alter according to the patient’s age, duration of the disease, and its severity but a range of physical and mental symptoms have been commonly reported. Prevalent complications include chronic diarrhea, abdominal pain, anemia, and growth problems in infancy and childhood as well as osteoporosis and cancer in later stages of life [10–13].

In this regard, self-care is defined as a set of conscious measures and activities taken by a person to lead a better life in the face of long-term physical or mental illnesses [14].. Rashid et al. [15] Investigated the impact of nutrition education on children with CD using new methods of education (video conference). The findings corroborated the participants’ satisfaction with the distance learning method due to its facilitative and understandable information transfer as well as reduced travel costs.

Self-care has been defined in terms of the recent advancements in the field of medical sciences, the increase in costs, the role of patients in their health status, and the lack of access to health and medical facilities by all members of the society. Educational needs have been introduced as the gap between the needed knowledge and performance in the current situation [16, 17]. The self-care training program covers areas, such as health maintenance, lifestyle modification, disease prevention, symptom assessment, disease treatment, and rehabilitation [18].

Dowd et al. [19] Also designed and launched a smartphone program to promote effective self-management of patients with CD and improve their intestinal health by providing rich educational information. The study was carried out in Canada according to the findings obtained from the patients’ needs assessment.

Mehraeen et al. [20] Concluded that identifying data requirements is the initial stage for designing an information system.

Annually, significant costs are spent on CD considering the costs of diagnosis and treatment as well as the patients’ and community members’ lack of information. Nowadays, self-care behaviors have been considered more emphatically due to the public health enhancement, emphasis on "prevention instead of treatment", as well as the role of people in controlling health and increasing awareness [21]. Along with the traditional training methods (in physical classes with groups of learners or one-on-one training), education based on new technology (e.g., smart cell phones, internet, etc.), generally called "Mobile Health", has received significant attention from officials and people in the society due to its ease of use, reduced cost, and availability [19]. Although self-care training programs can be an advancement in the field of CD care, no comprehensive self-care system is available for recording and organizing the information of Celiac patients in Iran. The present study was conducted given the increasing prevalence of CD in Iran; the need for identifying/validating data requirements, educating and improving public awareness and health at the community level; and the requirement for national information according to the genetic, cultural, nutritional, etiquette, and geographical conditions.

## Methods

The present research was carried out in two quantitative phases. The study was conducted in Dr. Shariati's educational, medical, and therapeutic center (at the Iranian Celiac Association) in Tehran as one of the largest and most equipped CD diagnosis centers in Iran that receive patients from all over the country with different cultural and geographical backgrounds.

### The first phase: determining the educational contents

In the first phase, the main categories and subcategories of the minimum required educational data were determined (through literature review) and organized in the form of a questionnaire.

Scientific references were selected from the materials published from April 2015 to April 2022 indexed in PubMed, ISC, and Springer databases using these keywords and their combinations: "application", "software", "celiac", "requirements", "minimum data", and "self-care". After analyzing and removing the duplicate articles, the main and detailed contents were obtained and organized into the form of a preliminary questionnaire.

The developed questionnaire included 105 items classified under nine topics: demographic information, clinical information of the respondent, long-term complications, tests, comorbidity, dietary recommendations, general recommendations from the viewpoints provided by the panel of experts, and technical capabilities. Initially, the questionnaire’s validity and reliability were confirmed (CVR = 0.81 & CVI = 0.89) via face and content validity of the questionnaire by Nineexperts in the fields of internal (*n* = 2), nutrition (*n* = 3), gastroenterology (*n* = 3), and medical informatics (*n* = 1). With regard to the questionnaire’s reliability, 15 experts (4 internists, 6 nutritionists, and 5 gastroenterologists) answered and filled the questionnaire. The KR-20 coefficient was calculated as 9. 0.

### The second phase: collecting the experts’ viewpoints and determining the final contents

Followed by preparing the questionnaire, the researcher had referred to the Celiac Association and the study hospital to collect data for two weeks. After obtaining written consent from all participants, the experts (present at the study location) were asked to complete the questionnaire so that the researcher could clarify and illuminate the items if needed. Data analysis was performed through descriptive statistics (mean and standard deviation) using SPSS software version 20. For data analysis, answers were weighted using dichotomous options of "unnecessary" (1 score) and "necessary" (2 scores). The educational content items that received an average score of 1.5 ≤ (more than 75% of the score) were selected as the necessary items. The prepared questionnaire was administered to 12 pundits selected purposively and non-randomly, who were required to determine the importance and necessity of the educational contents according to their background knowledge and type of expertise.

## Results

After summarizing the major and minor educational contents and systematizing them in the form of a questionnaire, 13 experts (Table 1) were needed to review and fill the questionnaire out. Based on the participants’ demographic characteristics, experts with 40 ≤ years (66.7%, *n* = 8) having work experience of 10 ≤ years (*n* = 7, 53%, 60%) had the highest frequency.

**Table 1:** The experts’ demographic information

Variable		*n*	%
Gender	Male	7	58.3
	Female	5	41.7
Age	< 30	0	0
	30–40	4	33.3
	> 40	8	66.7
Specialty	Nutrition	5	41.7
	Internist	4	33.43
	Gastroenterologist	3	25
Work Experience	< 10	3	41.7
	10–20	6	50
	> 20	1	8.3

In the researcher-made questionnaire, the educational needs of the Celiac self-care system were determined under nine major categories (demographic, clinical, long-term complications, comorbidity, tests, medicines, dietary recommendations, general recommendations, and technical capabilities) and 105 subcategories. Tables 2, 3, 4, 5, 6, 7, 8 and 9 include the frequency and percentage of educational content with regard to Celiac self-care needs. In the case of "demographic information", initial investigations of the literature provided 18 educational concepts, of which 14 were approved by the pundits. Consequently, the father's name, national code, number of children, and type of insurance were not considered necessary. All subcategories of the clinical information (20 items), including, tests (19 items), general recommendations (6 items), and technical capabilities (11 items)" were necessary by pundits. In the category of "medications and dietary recommendations", 15 subcategories were deemed necessary and only the sub-category "pure coffee without flavoring" was selected as unnecessary by the experts. Also, In the category of "long-term complications ", the sub-category " Gallbladder dysfunction " and In the category of " comorbidities", the sub-category " Colon Cancer" was selected as unnecessary by the experts. In the category "general recommendations in the field of dietary ", all subcategories (6 items) received the maximum attainable score (2 scores) indicating that all participants agreed upon their necessity. In the category entitled "technical capabilities", except for the subcategory "ability to provide instructions" with a mean score of 1.83 other subcategories obtained the highest attainable mean scores (2 points).

**Table 2:** The frequency of educational needs in the category of demographic information

Row	Information requirements	Agree		Disagree	
		*n*	%	*n*	%
1	First name, last name	12	100	0	0
2	Fathers’ name	2	16.7	10	83.3
3	Gender	12	100	0	0
4	ID number	4	33.3	8	66.7
5	Date of birth	12	100	0	0
6	Location of birth	12	100	0	0
7	Weight	12	100	0	0
8	Height	12	100	0	0
9	Blood group	8	66.7	4	33.3
10	Material status	12	100	0	0
11	Number of children	5	41.7	7	58.3
12	Type of insurance	3	25	9	75
13	History of CD in the family	12	100	0	0
14	BMI	12	100	0	0
15	Address	9	75	3	25
16	Telephone number	7	58.3	5	41.7
17	Date of visit	12	100	0	0
18	The number of hospitalization	11	91.7	1	8.3

**Table 3:** The frequency of educational needs in the category of clinical information

Row	Information requirements	Agree		Disagree	
		*n*	%	*n*	%
19	Child development disorder	10	83.3	2	16.7
20	Mouth sores	12	100	0	0
21	Irritable bowel syndrome	8	66.7	4	33.3
22	Peripheral neuropathy	10	83.3	2	16.7
23	B12 vitamin deficiency	10	83.3	2	16.7
24	Excretion of stomach gas	6	50	6	50
25	Chronic diarrhea	12	100	0	0
26	Constipation	10	83.3	2	16.7
27	Weight gain or loss	10	83.3	2	16.7
28	Feeling exhausted	8	66.7	4	33.3
29	Joint or bone pain	12	100	0	0
30	Iron deficiency anemia	10	83.3	2	16.7
31	Osteoporosis or decreased bone density	12	100	0	0
32	Behavioral changes	6	50	6	50
33	Menstrual disorder (Often due to excessive weight loss)	12	100	0	0
34	Infertility	10	83.3	2	16.7
35	Repeated miscarriage	12	100	0	0
36	Bleeding and hematoma (vitamin K deficiency)	11	91.7	1	8.3
37	Swelling of the oral mucosa and gums	12	100	0	0
38	Itchy skin lesions called dermatitis herpetiformis	12	100	0	0

**Table 4:** The frequency of educational needs in the category of long-term complications

Row	Information requirements	Agree		Disagree	
		*n*	%	*n*	%
39	Lack of vitamins and minerals	12	100	0	0
40	Central nervous system disorders	12	100	0	0
41	Colon Cancer	11	91.7	1	8.3
42	Gallbladder dysfunction	0	0	12	100
43	Occurrence of neurological disorders such as migraine, muscle coordination disorders, and insanity	10	83.3	2	16.7
44	Increased liver enzymes, liver disorder	12	100	0	0

**Table 5:** The frequency of educational needs in the category of comorbidities

Row	Information requirements	Agree		Disagree	
		*n*	%	*n*	%
45	Thyroid disease	12	100	0	0
46	Liver problems	12	100	0	0
47	Lupus	12	100	0	0
48	Rheumatoid Arthritis	12	100	0	0
49	Down syndrome	12	100	0	0
50	Type 1 diabetes	12	100	0	0
51	Osteoporosis	12	100	0	0
52	Colon Cancer	0	0	12	100
53	Occurrence of neurological disorders (migraine)	10	83.3	2	16.7

**Table 6:** The frequency of educational needs in the category of tests

Row	Information requirements	Agree		Disagree	
		*n*	%	*n*	%
54	FBS	10	83.3	2	16.7
55	Cholesterol	10	83.3	2	16.7
56	Tissue transglutaminase enzyme (TTGA) of IgA type	12	100	0	0
57	Anti-endomysial antibody (EMA)	12	100	0	0
58	Anti Gliadin Ab,IgG	12	100	0	0
59	Anti Gliadin Ab,IgA	12	100	0	0
60	C-Reactive Protein(CRP)	10	83.3	2	16.7
61	Endoscopy and sampling of the duodenum	12	100	0	0
62	Vitamin D	12	100	0	0
63	Thyroid tests TSH T3 T4	10	83.3	2	16.7
64	Ferritin	12	100	0	0
65	CBC	10	83.3	2	16.7
66	Stool Exam	12	100	0	0
67	TIBC	10	83.3	2	16.7
68	Alkalin Phosphatase	10	83.3	2	16.7
69	ZINC	10	83.3	2	16.7
70	IRON	12	100	0	0
71	AST(SGOT)	12	100	0	0
72	ALT(SGPT)	12	100	0	0

**Table 7:** Frequency of educational needs in the field of medications and dietary recommendations from the viewpoints of physicians

Row	Information requirements	Agree		Disagree	
		*n*	%	*n*	%
73	Gluten-free medicines	12	100	0	0
74	Gluten-free foods	12	100	0	0
75	Using bread made from rice and corn	12	100	0	0
76	Cereals and legumes	12	100	0	0
77	Fruits and vegetables	12	100	0	0
78	Meat and fish	12	100	0	0
79	Pure coffee without flavorings	4	33.3	8	66.7
80	Green tea	6	50	6	50
81	Nuts (almonds, pistachios, walnuts, hazelnuts)	12	100	0	0
82	Oils (corn oil, olive oil, sunflower oil)	12	100	0	0
83	Snacks (potato chips and roasted corn)	10	83.3	2	16.7
84	Rice	12	100	0	0
85	Potato	12	100	0	0
86	Gluten-free legumes and seeds	10	83.3	2	16.7
87	Types of gluten-free types of breads	10	83.3	2	16.7
88	Dairies	12	100	0	0

**Table 8:** Frequency of educational needs in the field of dietary recommendations from the viewpoints of physicians

Row	Information requirements	Agree		Disagree	
		*n*	%	*n*	%
89	Consuming fruits and vegetables 5^5^ times a day	12	100	0	0
90	Reduction of taking sugar and sweet foods	12	100	0	0
91	Consumption of plenty of fiber	12	100	0	0
92	Reduction of salt intake	12	100	0	0
93	Consuming liquids	12	100	0	0
94	Taking vitamin supplements	12	100	0	0

**Table 9:** Frequency of educational needs in the field of technical capabilities

Row	Information requirements	Agree		Disagree	
		*n*	%	*n*	%
95	Capability to remember taking medicines	12	100	0	0
96	Capability to remember adhering to the diet	12	100	0	0
97	Educational messages	12	100	0	0
98	Security requirements	12	100	0	0
99	Capability to remember exercising	12	100	0	0
100	Being user friendly	12	100	0	0
101	The ability to provide motivational messages	12	100	0	0
102	Being based on the web	12	100	0	0
103	Capability of reminding appointments	12	100	0	0
104	Capability of exchanging text messages	12	100	0	0
105	Ability of providing instructions	10	83.3	2	16.7

## Discussion

Considering the increased role of the patient in his health and the lack of a detailed and complete study for requirements needs assessment to design a self-care program for celiac patients, this study was made by a researcher questionnaire and based on the opinion of experts, educational needs were divided into 9 general sections with 105 sub-sections and It was approved by them.

. In fact, the educational need is defined as the gap between the current and the desired level of knowledge. Public training facilitates creating competence in people provided that the educational.program is designed and implemented based on the target community’s real needs. Identification of the patients’ educational needs is the first step toward promoting their skills and attitudes. Since educational resources are limited, care organizations should make a considerable effort in the field of educational needs assessment.

The studies conducted on CD in Iran were mostly focused on the disease prevalence in different regions and very few studies have dealt with the needed educational content.

Barzegar et al. [22] discussed the impact of education on the awareness of celiac patients, and the results showed that in most cases and educational topics, educational sessions increased the awareness of celiac patients. In the present study, an effort was made to examine and confirm the required and basic educational items separately and according to the opinions of different Murray experts.

In a similar study, Rashid et al. [15] and Mehraeen et al. [20] And Similar to the present research, the authors of this study also aimed to enhance the level of social awareness by employing technology-based educational methods and determining the appropriate educational content.

Another study by Barzegar et al. [23] was conducted with the aim of examining the knowledge of professional in the diagnosis and treatment of celiac disease. The results showed that the level of education is related to the work experience of professional, they both need additional training for diagnosis and treatment. In the present study, the work experience of the participants is at a favorable level. Also, the use of specialists is one of the weakness that is reported in the limitations section.

Dowd et al. [19] collected the pundits’ opinions regarding the most recent factors faced by these patients. Although this study is similar to the present investigation in many dimensions, In the same vein, our findings can be applied in designing application programs, minimum data, and registries. Haas et al. [24] Also presented an effective application and intervention program among celiac patients to improve their activation and quality of life. Buseck et al. [25] Reviewed blogs and websites related to CD to evaluate their information qualitatively and quantitatively. The findings revealed a large amount of unrelated information. Banti et al. [26] Not only checked the quality and validity of the information but also examined the quality of websites using the latest guidelines published by the European Society of Gastroenterology, Hepatology, and Pediatric Nutrition (ESPGHAN) and the World Gastroenterology Organization (WGO). As they concluded, the scrutinized websites conformed well to the above-mentioned standards. The present study was carried out based on these results to present the disease risk factors, updated information, and the required educational content with acceptable levels of validity and accuracy according to the pundits’ viewpoints.

Kiedrowski et al. [27] Examined Polish language videos available on YouTube for patients with CD. They noted that patients welcomed using YouTube as a source of additional knowledge. Basch et al. [28] Analyzed the English videos on YouTube about CD and delineated that most videos have been uploaded since 2010 and little information is available on the effects of age, the underlying disease, the impact of hereditary, and the stages of CD in children. The present study was conducted following the results of literature on the needed educational content and tried to revise the existing gaps as much as possible. So, all the necessary educational factors and content were collected from a variety of fields affecting CD and its subcategories.

## Limitations of the study

The prevalence of COVID-19 virus at the time of data collection, lack of time, and lack of some participants’ cooperation can be mentioned as probable limitations of our study. To meet the latter challenge, the researcher provided the pundits with comprehensive explanations about the study goals, advantages, and significance to persuade them to cooperate. Furthermore, the questionnaire was distributed electronically to facilitate accurate and fast data collection. Given the Coronavirus pandemic, only the experts’ opinions have been considered and only a limited number of respondents participated in the study So, future researchers are recommended to investigate the opinions of patients and medical center personnel in addition to the experts’ opinions. This study was conducted in Iran to expedite its development. Considering the influence of geographical location and genetics in patients with CD, future studies can be developed in West Asia.

## Conclusion

No standard and organized systems exist for information registration and management in developing countries, such as Iran because a wide range of information is produced with no comprehensive information recording systems. In the present study, we reviewed the related literature, developed a questionnaire, identified and validate the educational needs of celiac patients using a panel of experts, and classified the findings into nine categories. These requirements can be employed to design and create a self-care system for celiac patients and develop a systematic registry to record the data. Considering the lack of a self-care system for CD in Iran, planning and launching such a system at the national level can be a wise investment to improve the public’s level of awareness about this disease, provide patients with better services, and facilitate related research.

## Supplementary Information

**Additional file 1.**

## Data Availability

The data used and analysed during the current study are not publicly available due Iran University of Medical Sciences policy but are available from the corresponding author on reasonable request.

## Abbreviations

CD: Celiac Disease
WGO: World Gastroenterology Organization
ESPGHAN: European Society of Gastroenterology, Hepatology, and Pediatric Nutrition
CRP: C-Reactive Protein
