# Supplementary material for: Identifying and validating the educational needs to develop a Celiac Self-Care System
Source: BMC Prim Care. 2023 Jun 14;24:121. doi: 10.1186/s12875-023-02076-8 (PMC10265559; doi:10.1186/s12875-023-02076-8)
Supplement: Supplementary file 1 — Additional file 1. [file 12875_2023_2076_MOESM1_ESM.docx]

Dear respondents,

This questionnaire is designed to identify the necessary items for designing a conceptual model of self-care application for celiac patients. The questions are organized in different sections and have two Agree and Disagree answers.

Accurate response will help us to achieve research goals and correct results. The time to answer the questionnaire is about 25 to 30 minutes. If you have any questions, please contact Mr. Pakzad Rahmati. Thank you for your valuable time.

Pakzad Rahmati

Senior expert in health information technology

email: [pakzadrahmati.hit@gmail.com](mailto:pakzadrahmati.hit@gmail.com)

**A: Demographic Information**

Gender: Male Female

Age: <30 30-40 >40

Specialty: Nutrition Internist Gastroenterologist

Work Experience: <10 10-20 >20

B: Data Requirements items

B.1: Demographic Information

| Row | Information requirements | Agree | Disagree |
| --- | --- | --- | --- |
| 1 | First name, last name |  |  |
| 2 | Fathers’ name |  |  |
| 3 | Gender |  |  |
| 4 | ID number |  |  |
| 5 | Date of birth |  |  |
| 6 | Location of birth |  |  |
| 7 | Weight |  |  |
| 8 | Height |  |  |
| 9 | Blood group |  |  |
| 10 | Material status |  |  |
| 11 | Number of children |  |  |
| 12 | Type of insurance |  |  |
| 13 | History of CD in the family |  |  |
| 14 | BMI |  |  |
| 15 | Address |  |  |
| 16 | Telephone number |  |  |
| 17 | Date of visit |  |  |
| 18 | The number of hospitalization |  |  |

B.2: Clinical Information

| Row | Information requirements | Agree | Disagree |
| --- | --- | --- | --- |
| 19 | Child development disorder |  |  |
| 20 | Mouth sores |  |  |
| 21 | Irritable bowel syndrome |  |  |
| 22 | Peripheral neuropathy |  |  |
| 23 | B12 vitamin deficiency |  |  |
| 24 | Excretion of stomach gas |  |  |
| 25 | Chronic diarrhea |  |  |
| 26 | Constipation |  |  |
| 27 | Weight gain or loss |  |  |
| 28 | Feeling exhausted |  |  |
| 29 | Joint or bone pain |  |  |
| 30 | Iron deficiency anemia |  |  |
| 31 | Osteoporosis or decreased bone density |  |  |
| 32 | Behavioral changes |  |  |
| 33 | Menstrual disorder (Often due to excessive weight loss) |  |  |
| 34 | Infertility |  |  |
| 35 | Repeated miscarriage |  |  |
| 36 | Bleeding and hematoma (vitamin K deficiency) |  |  |
| 37 | Swelling of the oral mucosa and gums |  |  |
| 38 | Itchy skin lesions called dermatitis herpetiformis |  |  |

B.3: Long-Term Complications

| Row | Information requirements | Agree | Disagree |
| --- | --- | --- | --- |
| 39 | Lack of vitamins and minerals |  |  |
| 40 | Central nervous system disorders |  |  |
| 41 | Colon Cancer |  |  |
| 42 | Gallbladder dysfunction |  |  |
| 43 | Occurrence of neurological disorders such as migraine, muscle coordination disorders, and insanity |  |  |
| 44 | Increased liver enzymes, liver disorder |  |  |

B.4: Category Of Comorbidities

| Row | Information requirements | Agree | Disagree |
| --- | --- | --- | --- |
| 45 | Thyroid disease |  |  |
| 46 | Liver problems |  |  |
| 47 | Lupus |  |  |
| 48 | Rheumatoid Arthritis |  |  |
| 49 | Down syndrome |  |  |
| 50 | Type 1 diabetes |  |  |
| 51 | Osteoporosis |  |  |
| 52 | Colon Cancer |  |  |
| 53 | Occurrence of neurological disorders (migraine) |  |  |

B.5: Category Of Tests

| Row | Information requirements | Agree | Disagree |
| --- | --- | --- | --- |
| 54 | FBS |  |  |
| 55 | Cholesterol |  |  |
| 56 | Tissue transglutaminase enzyme (TTGA) of IgA type |  |  |
| 57 | Anti-endomysial antibody (EMA) |  |  |
| 58 | Anti Gliadin Ab,IgG |  |  |
| 59 | Anti Gliadin Ab,IgA |  |  |
| 60 | C-Reactive Protein(CRP) |  |  |
| 61 | Endoscopy and sampling of the duodenum |  |  |
| 62 | Vitamin D |  |  |
| 63 | Thyroid tests TSH T3 T4 |  |  |
| 64 | Ferritin |  |  |
| 65 | CBC |  |  |
| 66 | Stool Exam |  |  |
| 67 | TIBC |  |  |
| 68 | Alkalin Phosphatase |  |  |
| 69 | ZINC |  |  |
| 70 | IRON |  |  |
| 71 | AST(SGOT) |  |  |
| 72 | ALT(SGPT) |  |  |

B.6: Medications And Dietary Recommendations From The Viewpoints Of Physicians

| Row | Information requirements | Agree | Disagree |
| --- | --- | --- | --- |
| 73 | Gluten-free medicines |  |  |
| 74 | Gluten-free foods |  |  |
| 75 | Using bread made from rice and corn |  |  |
| 76 | Cereals and legumes |  |  |
| 77 | Fruits and vegetables |  |  |
| 78 | Meat and fish |  |  |
| 79 | Pure coffee without flavorings |  |  |
| 80 | Green tea |  |  |
| 81 | Nuts (almonds, pistachios, walnuts, hazelnuts) |  |  |
| 82 | Oils (corn oil, olive oil, sunflower oil) |  |  |
| 83 | Snacks (potato chips and roasted corn) |  |  |
| 84 | Rice |  |  |
| 85 | Potato |  |  |
| 86 | Gluten-free legumes and seeds |  |  |
| 87 | Types of gluten-free types of breads |  |  |
| 88 | Dairies |  |  |

B.7: Dietary Recommendations From The Viewpoints Of Physicians

| Row | Information requirements | Agree | Disagree |
| --- | --- | --- | --- |
| 89 | Consuming fruits and vegetables 5 times a day |  |  |
| 90 | Reduction of taking sugar and sweet foods |  |  |
| 91 | Consumption of plenty of fiber |  |  |
| 92 | Reduction of salt intake |  |  |
| 93 | Consuming liquids |  |  |
| 94 | Taking vitamin supplements |  |  |

B.8: Technical Capabilities

.

| Row | Information requirements | Agree | Disagree |
| --- | --- | --- | --- |
| 95 | Capability to remember taking medicines |  |  |
| 96 | Capability to remember adhering to the diet |  |  |
| 97 | Educational messages |  |  |
| 98 | Security requirements |  |  |
| 99 | Capability to remember exercising |  |  |
| 100 | Being user friendly |  |  |
| 101 | The ability to provide motivational messages |  |  |
| 102 | Being based on the web |  |  |
| 103 | Capability of reminding appointments |  |  |
| 104 | Capability of exchanging text messages |  |  |
| 105 | Ability of providing instructions |  |  |

C:Please enter your suggestions below

………………………………………………………………………………………………………………………………………………………………………………………………………………………………………………………………………………………………………………………………………………
